# Occluded vein as a predictor for complications in non-infectious transvenous lead extraction

**DOI:** 10.3389/fcvm.2022.1016657

**Published:** 2022-10-04

**Authors:** Anat Milman, Eran Leshem, Eias Massalha, Karen Jia, Amit Meitus, Saar Kariv, Yuval Shafir, Michael Glikson, David Luria, Avi Sabbag, Roy Beinart, Eyal Nof

**Affiliations:** ^1^Leviev Heart Institute, The Chaim Sheba Medical Center, Ramat Gan, Israel; ^2^Sackler School of Medicine, Tel Aviv University, Tel Aviv, Israel; ^3^The Jesselson Integrated Heart Center, Shaare Zedek Medical Center, Jerusalem, Israel; ^4^Hebrew University in Jerusalem Medical School, Jerusalem, Israel; ^5^Hadassah Medical Center, Heart Institute, Jerusalem, Israel

**Keywords:** transvenous lead extraction, non-infectious, occluded vein, complications, venography

## Abstract

**Background:**

The use of cardiovascular implantable electronic device (CIED) is steadily increasing, and complications include venous occlusion and fractured leads. Transvenous lead extraction (TLE) can facilitate the re-implantation of new leads.

**Aims:**

This study aims to explore predictors and complications of non-infectious TLE.

**Methods:**

This study involves a retrospective analysis and comparison of characteristics, complications, and outcomes of patients with and without occluded veins (OVs) undergoing TLE at our center.

**Results:**

In total, eighty-eight patients underwent TLE for non-infectious reasons. Indications for TLE were lead malfunction (62; 70.5%) and need for CIED upgrade (22; 25%). Fourteen patients referred due to lead malfunction had an OV observed during venography. The OV group (36 patients) were significantly older (65.7 ± 14.1 vs. 53.8 ± 15.9, *p* = 0.001) and had more comorbidities. Ejection fraction (EF) was significantly lower for the OV group (27.5 vs. 57.5%, *p* = 0.001) and had a longer lead dwelling time (3,226 ± 2,324 vs. 2,191 ± 1,355 days, *p* = 0.012). Major complications were exclusive for the OV group (5.5% vs. none, *p* = 0.17), and most minor complications occurred in the OV group as well (33.3 vs. 4.1%, *p* < 0.001). Laser sheath and mechanical tools for TLE were frequently used for OV as compared to the non-occluded group (94.4 vs. 73.5%, respectively, *p* = 0.012). Procedure success was higher in the non-occluded group compared to the OV group (98 vs. 83.3%, respectively, *p* = 0.047). Despite these results, periprocedural mortality was similar between groups.

**Conclusion:**

Among the TLE for non-infectious reasons, vein occlusion appears as a major predictor of complex TLE tool use, complications, and procedural success. Venography should be considered prior to non-infectious TLE to identify high-risk patients.

## What's new?

- Non-infectious causes are a prevalent indication for TLE in the current era. TLE is mainly performed due to lead malfunction and, to a lesser degree, due to the need for CIED upgrade.
- These subgroups of non-infectious TLE are inherently different, and the complication rate of TLE for CIED upgrade is considerably higher.
- The presence of an occluded vein is a major driving force for complex procedures, less procedural success, and abundant periprocedural complications.

## Introduction

The use of cardiovascular implantable electronic device (CIED) has expanded over the 4 decades, with over a million new CIEDs being implanted worldwide annually (1, 2). Although these devices have revolutionized the management of patients with arrhythmias and conduction disorders, they are associated with various infectious and non-infectious complications, which may necessitate their removal (1, 3, 4). The leads of CIEDs are the weakest link of the device, with lead failure rates estimated to occur at a rate of 0.29–0.45% annually (2, 5). The most common causes of lead removal include infection, venous occlusion, and mechanical lead failure (1).

Transvenous lead extraction (TLE) is completed in a stepwise manner starting with manual traction of the leads (with or without locking stylets), mechanical tools, and progressing to powered tools such as laser sheaths (6, 7). Despite advances in lead removal, current estimates from large multi-center reports suggest that a clinical failure of lead extraction rates ranges from 2.3 to 3.3%, and major adverse events during lead extraction occur in up to 1.8% of patients (6, 7). Considering the significant risk of complications related to lead removal, the benefit of the patient must outweigh any surgical risks. Less contention exists regarding the necessity of removing infected leads; however, the risk-benefit ratio for non-infected lead removal indications has conflicting evidence (1). Many non-infectious indications for lead removal are currently categorized as class II indications (1), and decisions to progress to TLE remain challenging. These indications include lead failure, chronic pain, non-functional leads in young patients, symptomatic venous stenosis or occlusion, and device upgrades (1, 4). Using a large tertiary referral retrospective single-center cohort, we aimed to analyze the indications, predictors, and outcomes of non-infectious lead removal.

## Materials and methods

### Study population

This was a retrospective, single-center cohort study of patients who underwent lead extraction for non-infectious causes between 2011 and 2020 at Sheba Medical Center, Israel, a referral tertiary hospital. The clinical and procedural data were gathered prospectively from the procedural report and patients' records. This study included 88 consecutive patients who underwent non-infectious lead extraction (a total of 146 leads), between January 2011 and March 2020. Patients who presented with any signs of infection such as fever or positive bacterial culture were excluded from the analysis. The decision to perform TLE in these cases was left to the discretion of the treating team and was performed in cases where abandoning leads was not clinically justified. The study was approved by the Local Institutional Review Board.

### TLE procedure

All TLE procedures were performed by qualified experienced operators with a cardiothoracic surgeon available on site. Procedures were performed under general anesthesia, with hemodynamic monitoring and transesophageal echocardiography (TEE). A large-bore femoral venous access was inserted in all patients in case femoral bailout was warranted and for the SVC bridging balloon wire after it became available. A stepwise approach was used in all patients as previously described by our group (8, 9). The TLE procedure was terminated after complete removal of the leads, when lead fragments could not be removed or in the event of a major complication.

Venography was routinely performed when a patient needed a CIED upgrade (22 patients) and in the rest by the discretion of the operator (26 patients). All patients without pre-procedural venography were retrospectively evaluated for vein occlusion by surveying the fluoroscopy data from the procedure. Some of the patients in the lead malfunction group had an occluded vein (OV) as observed by venography during the procedure (*n* = 14). We hypothesized that an OV is a significant contributor for the occurrence of complications during an extraction procedure, and thus, we regrouped all patients according to whether they were found to have an OV or not.

### Definitions

*Non-infectious* causes for lead removal were categorized as having an indication for TLE because of a lead malfunction, occluded vein, or other cause. The primary analysis focused on comparing the two major groups (lead malfunction and device upgrade in the setting of an OV).

*TLE procedures* were classified as s*imple* (when complete removal of leads was achieved with simple traction including the need for locking stylet) or *complex* (when the former failed and the operator proceeded to the mechanical tool, powered sheaths, laser sheath, or femoral approach using a snare or ablation catheter). Different *time points* were ascribed for the periods from first *device implantation to extraction* (representing the oldest lead dwelling time) and *last intervention to extraction* (elapsed time from last pocket intervention to extraction).

*Complications* were divided into *major complications* (defined as life-threatening as tamponade, required surgical intervention, or result in death). Complications that did not meet the major complication criteria were classified as *minor complications*.

*Success or failure* was defined by radiological success results and not clinical success results (10). Patients were divided depending on the outcome of the extraction procedure. Success was defined only if the complete removal of all leads (including “lead tips”) was achieved.

Patients were then regrouped according to the presence of an OV, regardless of whether the primary reason for non-infectious TLE was lead malfunction or device upgrade.

### Follow-up

Patients were followed up until 1 May 2021. Mortality status was updated from Israel's population registry, a national registry updated regularly.

### Statistical analysis

Categorical variables are reported in frequencies and percentages. All continuous variables were tested for normal distribution by the Kolmogorov-Smirnov test and by visualizing the Q–Q plot, plotting the distribution and variance of the residuals. Normally distributed continuous variables were reported as mean and standard deviation values, and differences between groups were assessed using the Student's *t*-test. Continuous variables not normally distributed were reported as median and interquartile range (IQR, 25th−75th percentiles) values, and significance was assessed using the Mann-Whitney U test or Kruskal Wallis test. All statistical tests were two-sided, and a *p* < 0.05 was considered significant.

A binomial multivariable logistic regression model analysis was employed to identify the predictors of the OV among TLE patients and the variables associated with periprocedural complication and mortality in patients undergoing TLE for non-infectious etiologies. The variables included in both models were prioritized based on statistical significance in the univariate analysis and those assumed to be clinically relevant based on previous publications and clinical plausibility.

Statistical analysis was performed using the SPSS software 27.0.0 (IBM, Armonk, NY, USA) and the R foundation statistical computing and graphics software (version 4.0.0).

## Results

### Study population

**Figure 1:**
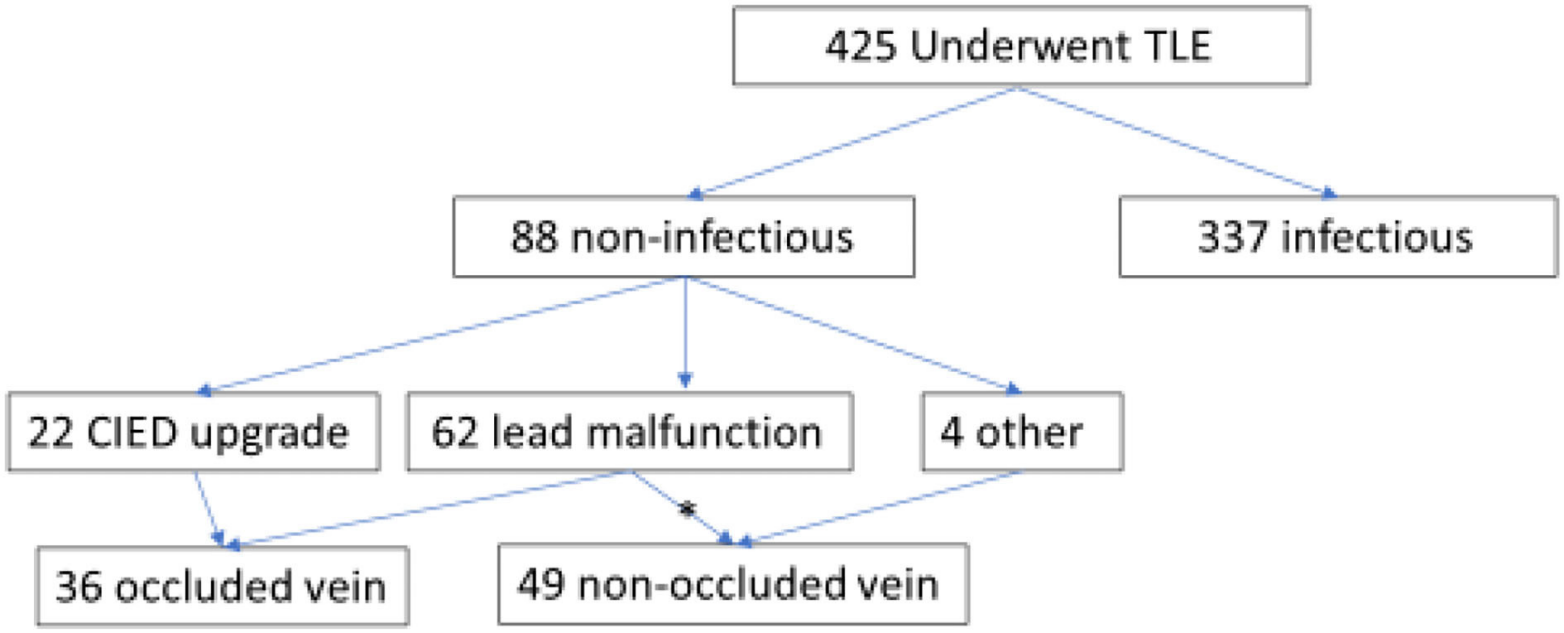
Study design. ^*^3 status of vein occlusion unknown.

During the trial period, 88 patients underwent TLE procedures at the Sheba Medical Center for non-infectious indications (out of a total of 425 TLE procedures performed) and were included in this cohort (Figure 1). The mean age at extraction was 58.4 ± 16.9 years, and the majority (75%) were male patients with a median ejection fraction (EF) of 35% [25, 60%]. In the majority of the cohort, one lead was extracted (52.3%). The TLE was defined as fully successful in most procedures (92%). Patient baseline characteristics are presented in Table 1.

**Table 1:** Patient characteristics of the entire cohort.

**All patients**	**Overall**	**Non-infectious**
Number of patients	425	88
Male sex	327 (76.9)	66 (75.0)
Age	65.5 ± 15.7	58.4 ± 16.8
Smoking	140 (32.9)	16 (18.2)
Atrial fibrillation	149 (35.1)	32 (36.4)
Hypertension	223 (52.5)	40 (45.5)
Heart failure	191 (44.9)	34 (38.6)
Stroke	54 (12.7)	8 (9.1)
Vascular disease	238 (56.0)	43 (48.9)
Malignancy	28 (6.6)	3 (3.4)
Diabetes mellitus	178 (41.9)	24 (27.3)
EF (%) [median (IQR)]	38.0 [25.0, 60.0]	35.00 [25.0, 60.0]
Creatinine (mg/dl)	1.05 [0.84, 1.50]	0.95 [0.80, 1.19]
Hemoglobin (mg/dl)	11.54 (2.13)	13.05 (1.73)
C-Reactive protein (mg/dl)	24.70 [6.91, 74.97]	6.07 [1.88, 15.97]
Albumin (g/dl)	3.44 (0.80)	3.96 (0.52)
**Device type**		
CRT-D	108 (25.4)	19 (21.6)
CRT-P	11 (2.6)	3 (3.4)
ICD	139 (32.7)	40 (45.4)
PM	166 (39.1)	26 (29.5)
**Number extracted leads**		
1	105 (24.7)	46 (52.3)
2	186 (43.8)	29 (33.0)
3	101 (23.8)	13 (14.8)
**Extraction success**		
Full	390 (91.8%)	81 (92%)
Partial	21 (4.9%)	2 (2.3%)
Failure	14 (3.3%)	5 (5.7%)

### Extraction indication

The majority were extracted due to a non-functioning lead (70.5%). Further indications included device upgrade (25%) and four patients (4.5%) because of other causes (heart transplant, intractable pain, irradiation, and severe tricuspid regurgitation).

### Lead malfunction compared to device upgrade

Table 2 provides the primary comparison of the two major groups of non-infectious indication for TLE for characteristics, complications, and extraction methods.

**Table 2:** Comparison of TLE due to lead malfunction or device upgrade in the setting of an occluded vein.

	**Device upgrade**	**Lead malfunction**	***P*-Value**
Number of patients	22	62	
Male	16 (72.7)	48 (77.4)	0.879
Age	68.3 ± 12.6	55 ± 17.2	0.002
Smoking	3 (13.6)	13 (21.0)	0.4
Atrial fibrillation	10 (45.5)	22 (35.5)	0.533
Hypertension	14 (63.6)	25 (40.3)	0.13
Heart failure	8 (36.4)	25 (40.3)	0.509
Stroke	3 (13.6)	5 (8.1)	0.171
Vascular disease	15 (68.2)	27 (43.5)	0.022
Malignancy	1 (4.5)	1 (1.6)	0.174
Diabetes mellitus	8 (36.4)	15 (24.2)	0.476
EF (%) [median (IQR)]	25.00 [22.50, 30.00]	52.50 [35.00, 60.00]	<0.001
Creatinine (mg/dl)	1.25 ± 0.4	0.98 ± 0.4	0.005
Hemoglobin (mg/dl)	12.6 ± 1.6	13.2 ± 1.7	0.138
C-Reactive protein (mg/dl)	7.7 ± 10.5	17.2 ± 22.8	0.302
Albumin (g/dl)	3.8 ± 0.4	4 ± 0.5	0.086
Referral from other hospital	17 (77.3)	39 (62.9)	0.334
**Device type**			0.176
CRT-D	4 (18.2)	14 (22.6)	
CRT-P	2 (9)	1 (1.6)	
ICD	8 (36.4)	31 (50)	
PM	8 (36.4)	16 (25.8)	
**Number extracted leads**			0.314
1	9 (40.9)	37 (59.7)	
2	9 (40.9)	17 (27.4)	
3	4 (18.2)	8 (12.9)	
First device implant to extraction (days)	2,712.95 ± 1,803	2,545.6 ± 1,917.5	0.722
Last intervention to extraction (days)	1,454.2 ± 901.8	1,457.2 ± 970.6	0.99
**Extraction type**			0.21
Simple	2 (9.1)	14 (22.6)	
Complex	20 (90.9)	48 (77.4)	
**Extraction success**			0.54
Full	20 (90.9)	57 (91.9)	
Partial	0	2 (3.2)	
Failure	2 (9.1)	3 (4.8)	
Minor complication	9 (40.9)	5 (8.1)	<0.001
Major complication	2 (9.1)	0 (0.0)	0.06
Periprocedural death	2 (5.5)	1 (2)	0.38
Follow up time (days)	989 [353, 2,219]	1,365 [416, 2,259]	0.36
Death during follow up	5 (22.7)	7 (11.3)	0.363

The TLE patients for device upgrade were significantly older than those with a lead malfunction (mean age 68.3 ± 12.6 vs. 55 ± 17.2, respectively, *p* = 0.002), had more vascular disease (68.2 vs. 43.5%, respectively, *p* = 0.022), had a higher creatinine level (1.25 ± 0.4 vs. 0.98 ± 0.4 mg/dl, respectively, *p* = 0.005), and had a lower EF (25 vs. 52.5%, respectively, *p* < 0.001). No difference was observed in the type of extracted device or number of leads, as well as lead dwelling time.

The extraction method used did not differ between these two groups, as well as the success rate of the procedure.

There were significantly more major and minor complications in the CIED upgrade group (minor 40.9 vs. 8.1%, respectively, *p* < 0.001 and major 9.1% vs. none, respectively, *p* = 0.06). One patient undergoing TLE for device upgrade perished during the procedure (due to SVC tear). Overall death during follow-up did not differ between the groups.

### Occluded vein presence

We observed 14/62 patients (22.6%) from the lead malfunction group with an OV during the procedure. A new total of 36 patients in our cohort had an OV and were compared to 49 patients without OV. In 3/88 patients, data on OV could not be retrieved. These groups were compared accordingly as shown in Table 3.

**Table 3:** Comparison according to occluded vein presence.

	**No vein occlusion**	**Occluded vein**	***P*-Value**
Number of patients	49	36	
Male	37 (75.5)	27 (75.0)	1
Age	53.84 ± 15.91	65.69 ± 14.14	0.001
Smoking	9 (18.4)	7 (19.4)	0.471
Atrial fibrillation	12 (24.5)	19 (52.8)	0.018
Hypertension	15 (30.6)	24 (66.7)	0.003
Heart failure	17 (34.7)	17 (47.2)	0.205
Stroke	3 (6.1)	4 (11.1)	0.345
Vascular disease	20 (40.8)	22 (61.1)	0.07
Malignancy	2 (4.1)	1 (2.8)	0.481
Diabetes mellitus	11 (22.4)	13 (36.1)	0.286
EF (%) [median (IQR)]	57.5 [35, 60]	27.5 [25, 37.25]	0.001
Creatinine (mg/dl)	1.02 (0.39)	1.10 (0.36)	0.395
Hemoglobin (mg/dl)	13.56 (1.77)	12.54 (1.52)	0.01
C-Reactive protein (mg/dl)	16.39 (19.87)	12.61 (22.85)	0.627
Albumin (g/dl)	4.13 (0.52)	3.79 (0.48)	0.005
Referral from other hospital	28 (57.1)	28 (77.8)	0.08
**Device type**			0.187
CRT-D	11 (22.4)	8 (22.2)	
CRT-P	1 (2.1)	2 (5.6)	
ICD	26 (53.1)	12 (33.3)	
PM	11 (22.4)	14 (38.9)	
First device to extraction (days)	2,191 ± 1,355	3,226 ± 2,324	0.012
Last intervention to extraction (days)	1,438 ± 1,010	1,584 ± 981	0.528
**Extraction type**			0.012
Simple	13 (26.5)	2 (5.6)	
Complex	36 (73.5)	34 (94.4)	
**Number of leads extracted**			0.202
1	30 (61.2)	15 (41.7)	
2	13 (26.5)	14 (38.9)	
3	6 (12.2)	7 (19.4)	
**Extraction success**			0.047
Full	48 (98)	30 (83.3)	
Partial	0	2 (5.6)	
Failure	1 (2)	4 (11.1)	
Minor complication	2 (4.1)	12 (33.3)	<0.001
Major complication	0 (0.0)	2 (5.5)	0.17
Periprocedural death	1 (2)	2 (5.5)	0.38
Follow up time (days)	1,325 [432, 2,269]	1,218 [386, 2,053]	0.36
Death during follow up	6 (12.5)	7 (19.4)	0.38

Patients with an OV were significantly older (65.7 ± 14.1 vs. 53.8 ± 15.9, respectively, *p* = 0.001). Both groups had a majority of male patients undergoing TLE.

The OV group had a higher comorbidity prevalence such as atrial fibrillation (52.8% vs. 24.5% for the non-occluded group, *p* = 0.018), hypertension (66.7 vs. 30.6%, *p* = 0.003), and a trend toward more vascular disease (61.1 vs. 40.8%, *p* = 0.07). EF was significantly lower for the OV group (27.5 vs. 57.5% for the non-occluded group, *p* = 0.001).

The OV group had a lower hemoglobin (12.5 ± 1.5 vs. 13.6 ± 1.8 mg/dl, *p* = 0.01) and a lower albumin (3.8 ± 0.5 vs. 4.13 ± 0.5 g/dl, *p* = 0.005).

There was no difference in the type of CIED extracted or the number of leads. Lead dwell time in the OV group was longer compared to the non-occluded group (3,226 ± 2,324 vs. 2,191 ± 1,355 days, respectively, *p* = 0.012).

**Figure 2:**
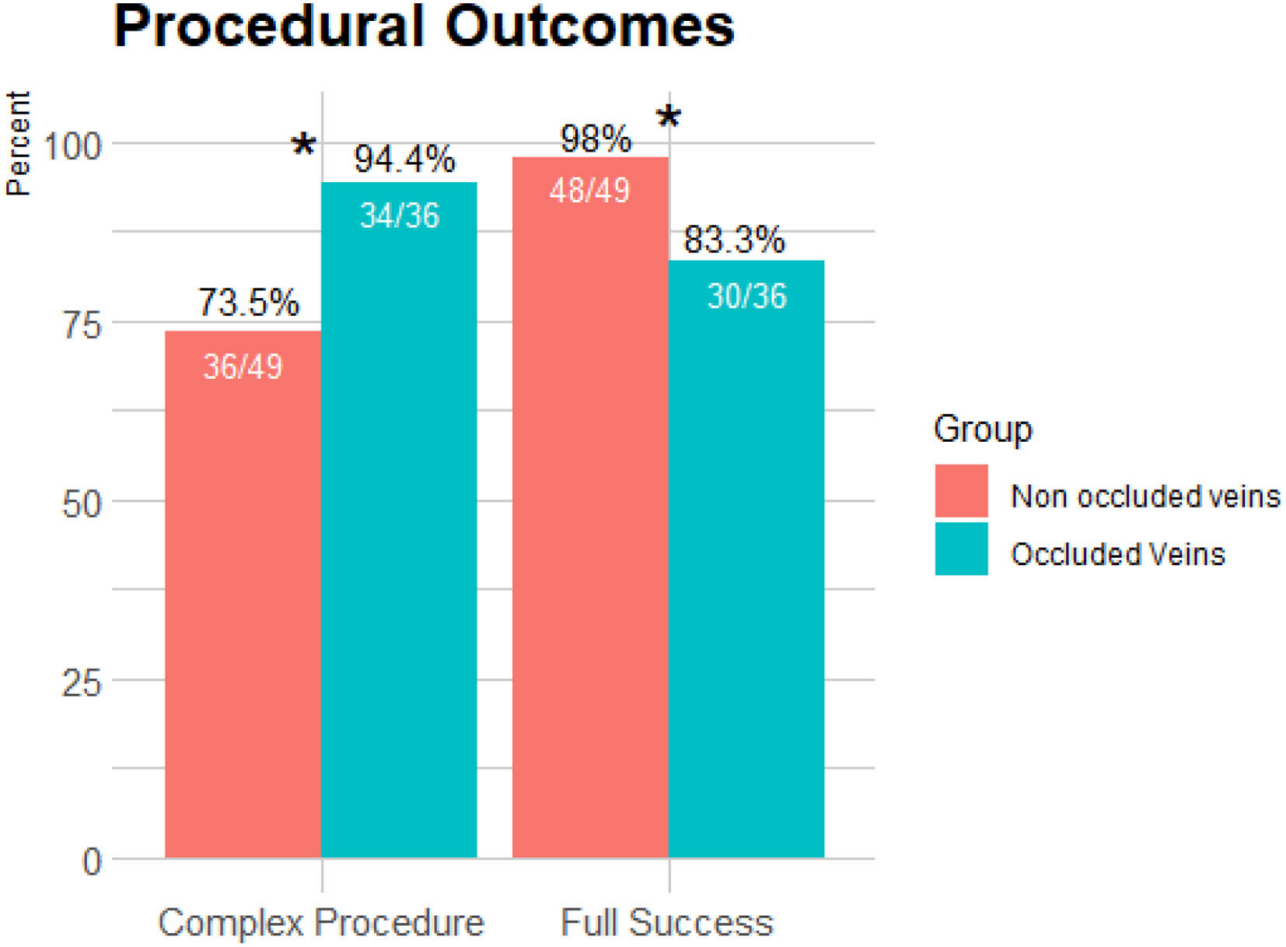
Comparison of procedural outcomes between occluded and non-occluded vein groups. More complex methods were used for TLE in the presence of an occluded vein as compared to the non-occluded group (*p* = 0.012). Radiological success was more common in the non-occluded group compared to the occluded vein group (*p* = 0.047). *p < 0.05.

The methods used for TLE were significantly different between the groups with more complex methods used for OV as compared to the non-occluded group (94.4 vs. 73.5%, respectively, *p* = 0.012). Radiological success was more common in the non-occluded group compared to the OV group (98 vs. 83.3%, respectively, *p* = 0.047; Figure 2).

**Figure 3:**
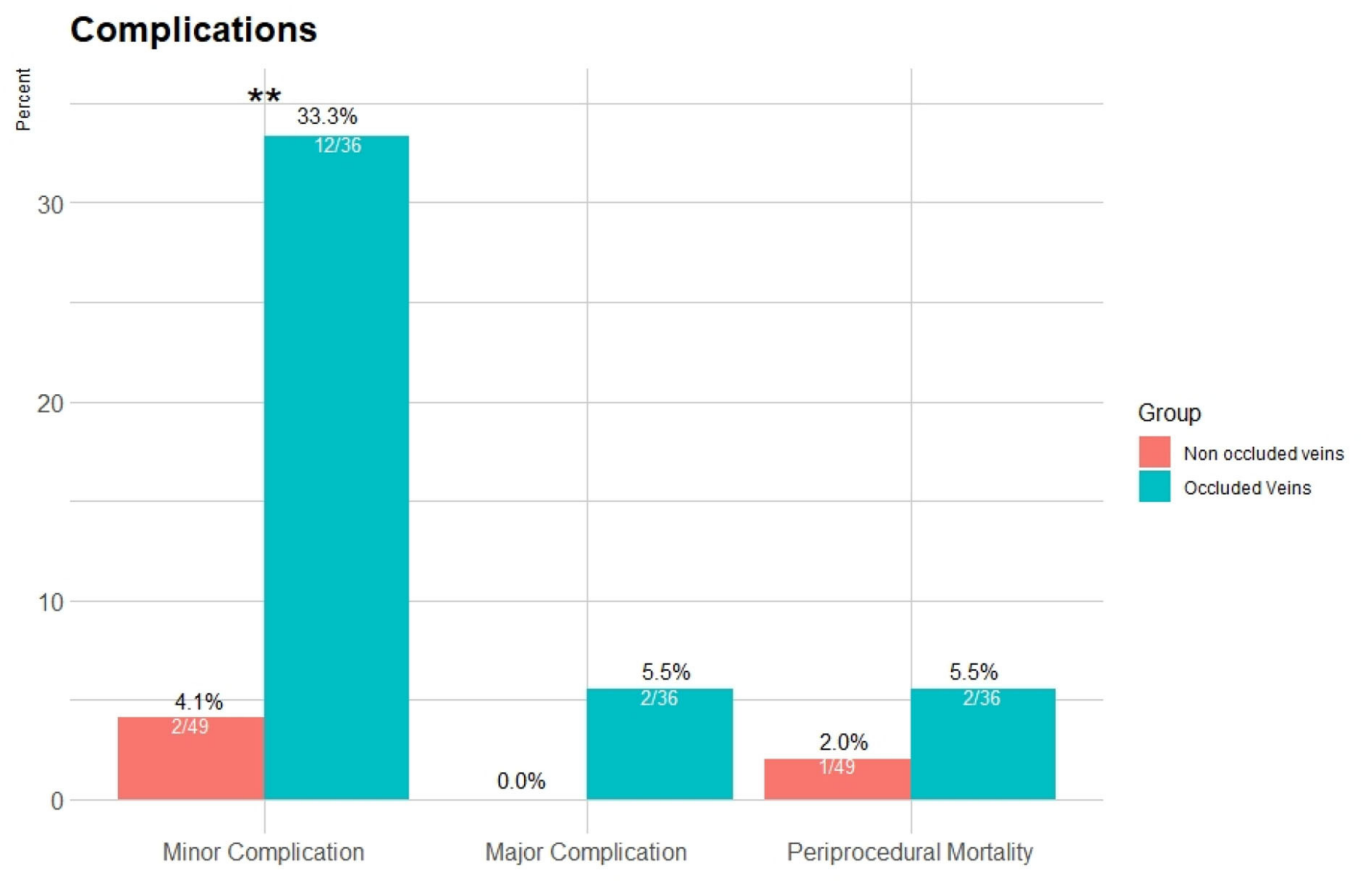
Complication rates for the occluded and non-occluded vein groups. Major complications were exclusive for the occluded vein group (2 patients), and minor complications were more frequent (*p* < 0.001). Periprocedural death during follow-up was similar (*p* = 0.38). **p < 0.001.

Major complications were exclusively found in the OV group (two patients compared to none in the non-occluded vein, *p* = 0.17), and minor complications were more frequent in the OV group as well (33.3 vs. 4.1%, *p* < 0.001; Figure 3).

Periprocedural death during follow-up was similar (2% in the non-occluded vein group vs. 5.5% in the occluded group, *p* = 0.38; Figure 3).

A binomial multivariable logistic regression model was deployed to identify the clinical parameters associated with the presence of an OV. Older age [odds ratio (OR): 1.07 (95% CI: 1.01–1.13 per 1 year), *p* = 0.01] and hypertension [OR: 5.79 (95% CI: 1.68–20.08, *p* < 0.01)] were shown to be strongly correlated with vein occlusion. Lower left ventricular ejection fraction (LVEF) and the time elapsed from implantation to extraction were predictors for vein occlusion (Table 4).

**Table 4:** Multivariate analysis of predictors for occluded veins.

**Predictors**	**Univariate analysis**			**Multivariable analysis**		
	**Odds ratio**	**Confidence interval**	***P*-Value**	**Odds ratio**	**Confidence interval**	***P*-Value**
Age	*1.05*	*1.02–1.09*	*0.002*	*1.07*	*1.01–1.13*	*0.018*
Atrial fibrillation	*3.2*	*1.29–1.83*	*0.01*	*1.49*	*0.43–5.07*	*0.52*
HTN	*4.12*	*1.65–10.29*	*0.002*	*5.79*	*1.67–20.08*	*0.006*
LVEF	*0.95*	*0.92–0.98*	*0.001*	*0.95*	*0.91–0.99*	*0.016*
Years (from first implantation to extraction)	*1.12*	*1.02–1.25*	*0.019*	*1.21*	*1.07–1.36*	*0.003*

HTN, hypertension; LVEF, left ventricular ejectin fraction.

A binomial multivariable backward regression model was performed to ascertain the predictors for periprocedural complications, including mortality. Patients with an OV had significantly higher odds for periprocedural complications, including mortality [OR: 15.08 (95% CI: 2.76–82.2, *p* < 0.01) Table 5].

**Table 5:** Multivariable regression (backward Wald) models of predictors for periprocedural complications and mortality.

**Predictors**	**1st step model**			**Final step model**		
	**Odds ratio**	**Confidence interval**	***P*-Value**	**Odds ratio**	**Confidence interval**	***P*-Value**
Age	*0.98*	*0.93–1.04*	*0.54*			
Male gender	*1.4*	*0.22–9.5*	*0.72*			
Atrial fibrillation	*0.13*	*0.02–0.81*	*0.29*	*2.4*	*0.06–1.01*	*0.052*
HTN	*4.8*	*0.98–23.8*	*0.052*	*3.60*	*0.84–15.4*	*0.084*
LVEF < 40%	*0.50*	*0.74–3.40*	*0.48*			
Years (from first implantation to extraction)	*1.09*	*0.95–1.26*	*0.21*			
Occluded vein	*15.03*	*1.86–121.8*	*0.11*	*15.08*	*2.76–82.2*	*0.002*

HTN, hypertension; LVEF, left ventricular ejectin fraction.

## Discussion

Main findings of our study are as follows:

- TLE for non-infectious causes is a prevalent indication for TLE in the current era and is mainly performed due to lead malfunction and, to a lesser degree, due to the need for CIED upgrade.
- These subgroups of non-infectious TLE are inherently different, and the complication rate of TLE for CIED upgrade is considerably higher.
- Analysis according to the presence of an OV reveals that this factor is a major driving force for complex procedures, less procedural success, and abundant periprocedural complications.
- The present retrospective analysis of 88 consecutive patients undergoing TLE for non-infectious reasons emphasizes the importance of pre-procedural planning and venography to identify those with an OV, as these patients were found to have a worse outcome.

### Non-infectious TLE

Patients referred for TLE for non-infectious reasons comprise a significant portion of those undergoing the procedure (6); however, they are underrepresented in the literature, and indications for appropriate extraction are all currently a class 2 indication (1).

In the ELECTRa European prospective cohort, the rate of TLE for a non-infectious etiology was found to be 47.3% of all TLE procedures (1,683/3,555) (6). The recently published CLEAR registry including eight Canadian centers showed that non-infectious reasons for TLE were the majority of cases, with only 48.6% of TLEs due to an infection (11). A recent study from the United Kingdom included a total of 1,151 patients, with 632 (54.9%) and 519 (45.1%) patients representing infective and non-infective indications, respectively (12).

Our cohort of 88 such patients represents a non-negligible fraction (21%), which is less than the aforementioned rates of non-infectious TLE published. However, in Mayo Clinic, out of the 480 TLE performed between January 2001 and October 2012, 123 procedures (25.6%) were because of superfluous leads (13), and indications for extraction were malfunction in 41%, recall in 26%, and upgrade in 15% (13). Archontakis et al. (14) provided data from their high-volume reference center for CIED extractions in Athens, with a total of 242 consecutive patients undergoing TLE of which a minority was for non-infectious reasons (16.9%). This difference may be due to a strict adherence to current indications and a higher tendency to add leads in cases of lead malfunction of required upgrade.

### Occluded veins

Patients with an OV were older and had more comorbidities such as atrial fibrillation, hypertension, and a borderline tendency for vascular disease. Not surprisingly, these patients also had a lower EF, as most of these patients were referred for a CIED upgrade. Our secondary analysis divided patients by results of venography, comparing the presence of OVs and the effect on the TLE procedure and outcome.

### Predictors for occluded veins

Our findings reveal that advanced age, hypertension, decreased LVEF, and lead dwell time were significant predictors for an OV that will result in a more complex procedure with less favorable outcomes.

An important predictor for vein occlusion was lead dwell time, which was significantly longer in the OV group compared to those without an occlusion. This was also observed by Pieper et al. (15), when assessing venous obstruction in patients undergoing revision of CIED. The issue of the number of leads as a risk factor for an OV is debatable. The existence of multiple leads has previously been found to be a risk factor for developing venous thrombosis (16, 17), and thus, complete lead extraction is recommended whenever there are more than four leads on one side or five leads through the superior vena cava (SVC) (16). On the contrary, some studies failed to show a correlation between venous complications and lead burden (18, 19) in accordance with our findings. Moreover, Li et al. (19) found that infection (both systemic and local infection) and not the number of leads was associated with an increased risk of venous occlusion.

### Results of OV procedures

The most clinically important findings of our analysis were the significantly more difficult procedures performed in the presence of OV, as reflected by the need to use more complex methods and the lower full success rate of the procedure. Complication rates were more frequent in the aforementioned group as well. Li et al. (19) demonstrated, although in a cohort of infected devices, that lead extraction was more challenging in patients with venous occlusion, requiring superior devices and longer time. Opposed to these findings, Boczar et al. (20) could not observe vein occlusion to influence the effectiveness, safety, and the use of additional devices during TLE procedures; however, their population was a mix of infected and non-infected devices, with a significantly higher rate of vein occlusion (36.1%).

### Limitations

We acknowledge several limitations. This was a single-center retrospective cohort, and the results as such may not be generalized to other populations. Performing a TLE in these cases was left to the discretion of the treating physician team as was the decision to perform a pre-procedural venography. Of note, not all lead malfunction procedures had a venography prior to the procedure, mainly due to the fact that it was determined to proceed with TLE regardless of the venography results. No attempted venoplasty was performed in cases of venous occlusion, and these were referred for TLE.

## Conclusion

The TLE for non-infectious reasons is common, and strict criteria and indications should be formalized. Vein occlusion appears as a major predictor for complex TLE tools use, complications, and procedural success. Venography should be considered prior to non-infectious TLE to identify venous occlusion and high-risk patients.

## Data availability statement

The raw data supporting the conclusions of this article will be made available by the authors, without undue reservation.

## Ethics statement

The studies involving human participants were reviewed and approved by The Chaim Sheba Medical Center, Tel Hashomer, Israel. The patients/participants provided their written informed consent to participate in this study.

## Author contributions

AMi, EL, and EN contributed to conception and design of the study. AMi, KJ, SK, and YS organized the database. EM performed the statistical analysis. AMi and EL wrote the first draft of the manuscript. KJ wrote sections of the manuscript. All authors contributed to manuscript revision, read, and approved the submitted version.

## Conflict of interest

The authors declare that the research was conducted in the absence of any commercial or financial relationships that could be construed as a potential conflict of interest.

## Publisher's note

All claims expressed in this article are solely those of the authors and do not necessarily represent those of their affiliated organizations, or those of the publisher, the editors and the reviewers. Any product that may be evaluated in this article, or claim that may be made by its manufacturer, is not guaranteed or endorsed by the publisher.
